# Molecular structures and functional exploration of NDA family genes respond tolerant to alkaline stress in *Gossypium hirsutum* L.

**DOI:** 10.1186/s40659-022-00372-8

**Published:** 2022-01-21

**Authors:** Yapeng Fan, Yuexin Zhang, Cun Rui, Hong Zhang, Nan Xu, Jing Wang, Mingge Han, Xuke Lu, Xiugui Chen, Delong Wang, Shuai Wang, Lixue Guo, Lanjie Zhao, Hui Huang, Junjuan Wang, Liangqing Sun, Chao Chen, Wuwei Ye

**Affiliations:** State Key Laboratory of Cotton Biology / Institute of Cotton Research of Chinese Academy of Agricultural Sciences / Zhengzhou Research Base, School of Agricultural Sciences, Zhengzhou University Research Base, Zhengzhou University / Key Laboratory for Cotton Genetic Improvement, MOA, Anyang, 455000 Henan China

**Keywords:** NDA, Phylogenetic analysis, *Cis*-elements, Expression pattern, VIGS, Alkaline stress

## Abstract

**Background:**

The internal NAD(P)H dehydrogenase (NDA) gene family was a member of the NAD(P)H dehydrogenase (ND) gene family, mainly involved in the non-phosphorylated respiratory pathways in mitochondria and played crucial roles in response to abiotic stress.

**Methods:**

The whole genome identification, structure analysis and expression pattern of NDA gene family were conducted to analyze the NDA gene family.

**Results:**

There were 51, 52, 26, and 24 NDA genes identified in *G. hirsutum*, *G. barbadense*, *G. arboreum* and *G. raimondii*, respectively. According to the structural characteristics of genes and traits of phylogenetic tree, we divided the NDA gene family into 8 clades. Gene structure analysis showed that the NDA gene family was relatively conservative. The four *Gossypium* species had good collinearity, and segmental duplication played an important role in the evolution of the NDA gene family. Analysis of *cis*-elements showed that most *GhNDA* genes contained *cis*-elements related to light response and plant hormones (ABA, MeJA and GA). The analysis of the expression patterns of *GhNDA* genes under different alkaline stress showed that *GhNDA* genes were actively involved in the response to alkaline stress, possibly through different molecular mechanisms. By analyzing the existing RNA-Seq data after alkaline stress, it was found that an NDA family gene *GhNDA32* was expressed, and then the*GhNDA32* was silenced by virus-induced gene silencing (VIGS). By observing the phenotype, we found that the wilting degree of silenced plants was much higher than that of the control plant after alkaline treatment, suggesting that *GhNDA32* gene was involved in the response to alkaline stress.

**Conclusions:**

In this study, *GhNDAs* participated in response to alkaline stress, especially NaHCO_3_ stress. It was of great significance for the future research on the molecular mechanism of NDA gene family in responding to abiotic stresses.

**Supplementary Information:**

The online version contains supplementary material available at 10.1186/s40659-022-00372-8.

## Introduction

With the continuous increase in the area of salt-alkaline land worldwide, salt-alkaline stress had become an important abiotic stress that restricted plant growth and development. Salt-alkaline stress was divided into salt stress and alkaline stress. Salt stress was mainly caused by neutral salts such as NaCl and Na_2_SO_4_, and alkaline stress was usually caused by Na_2_CO_3_ and NaHCO_3_ [1]. Previous studies found that alkaline stress was more harmful than salt stress [2]. At present, little was known about the mechanism of crop tolerance to alkaline stress. Therefore, it was very important to explore the mechanism of alkaline stress.

Mitochondria were an important place for energy production in plants, previous research found that plant mitochondria contained a complex electron transport chain, in which there was an alternative respiratory pathway (AP), and the difference of the AP was that it was not linked to energy conservation [3]. It was well known that most of the energy produced by plants come from mitochondria, at the meantime, plant mitochondria were also a source of reactive oxygen species (ROS), studies showed that the presence of the AP could prevent over-reduction of the electron transport chain (ETC), thereby minimizing the generation of ROS and protecting plants from oxidative damage [4, 5, 6]. In addition, AP actively participated in the response to abiotic stress, such as temperature, nutrients, heavy metals, high light, drought and oxidative stress [7].

AP contained two important components, the alternative oxidase (AOX) and type II NAD(P)H dehydrogenases (NDs). Expression of the AOX genes was highly responsive to abiotic and biotic stress, as well as dysfunctions in respiratory metabolism [8], and ROS may be an important signal controlling AOX expression [9]. In plants, fungi and some certain bacteria, there were many type II NAD(P)H dehydrogenases (NDs), which were small single proteins attached to either side of the inner membrane [10]. In potato, *St*-*nda1* gene was shown to be tightly bound to the inner surface of the mitochondrial inner membrane, while *St*-*ndb1* was loosely bound to the outer mitochondrial membrane [11, 12]. In *Arabidopsis*, the NDs could be divided into three subgroups, *AtNDA* (1, 2), *AtNDB* (1-4), and *AtNDC1*, *AtNDA1*, *2* and *AtNDC1* were identified as encoding internal NAD(P)H dehydrogenases, whereas *AtNDB1*-*4* encode external NAD(P)H dehydrogenases [13, 14].

NDA genes, belonged to the subclass of NDs, played a vital role in response to abiotic stress. Studies found that they were involved in the response to light, cold and Pi stress. The expression of *nda1* in *Arabidopsis* was dependent on light and circadian regulation, which played an important role in the photosynthesis and other respiratory NADH oxidation [14]. In potato, the immunodetectable NDA1 protein abundance and the internal rotenone insensitive NADH dehydrogenase activity were all affected by light, suggesting that the *nda1* gene was involved in photosynthetically associated processes, most likely photorespiration [15]. After cold treatment, potato leaves could induce the production of oxidase and ROS, and the expression of NDA1 gene decreased, which may be related to the redox function of NDA expression [16]. Previous research found that low-level expression of *NDAs* could lead to the delayed growth and restrict citric acid cycle reactions but apparently had no effect on photosynthesis [17]. In *Arabidopsis*, a non-phosphorylating mitochondrial electron transport chain consisting of *NDA2*, *NDB2* and *AOX* was synthesized to maintain respiratory electron flow through the mitochondrial electron transport chain during Pi stress [18]. However, whether NDA family genes responded to alkaline stress in cotton had not been reported, so it was meaningful to study it.

Cotton is an important fiber crop and a model crop for studying polyploidy and evolution [19]. *Gossypium hirsutum* (*G. hirsutum*) and *Gossypium barbadense* (*G. barbadense*) are the two most widely cultivated allotetraploid cotton species in the world, which are formed by inter-genomic hybridization of *Gossypium arboreum* (*G. arboreum*) and *Gossypium raimondii* (*G. raimondii*) [20]. Cotton is also a pioneer crop in salt-alkaline land, with a certain degree of salt-alkali tolerance, and is a common crop for us to study salt-alkaline stress [21]. Exploring the alkaline stress response genes will help promote the cotton’s tolerance to alkaline stress. From the RNA-Seq data, an NDA gene *GhNDA32* was found to respond to alkaline stress, so it is necessary to analyze its phylogenetic relationship and study the alkaline stress tolerance of the family. This study aimed to explore gene structure, evolutionary relationship, expression pattern, and *cis*-acting elements of NDA family. An NDA gene belonged to clade a, *GhNDA32*, was isolated and characterized, and we performed a preliminary analysis of the expression pattern of *GhNDA32*. This study provided potential candidate genes for cotton gene functional verification.

## Results

### Identification of NDA proteins

**Fig. 1:**
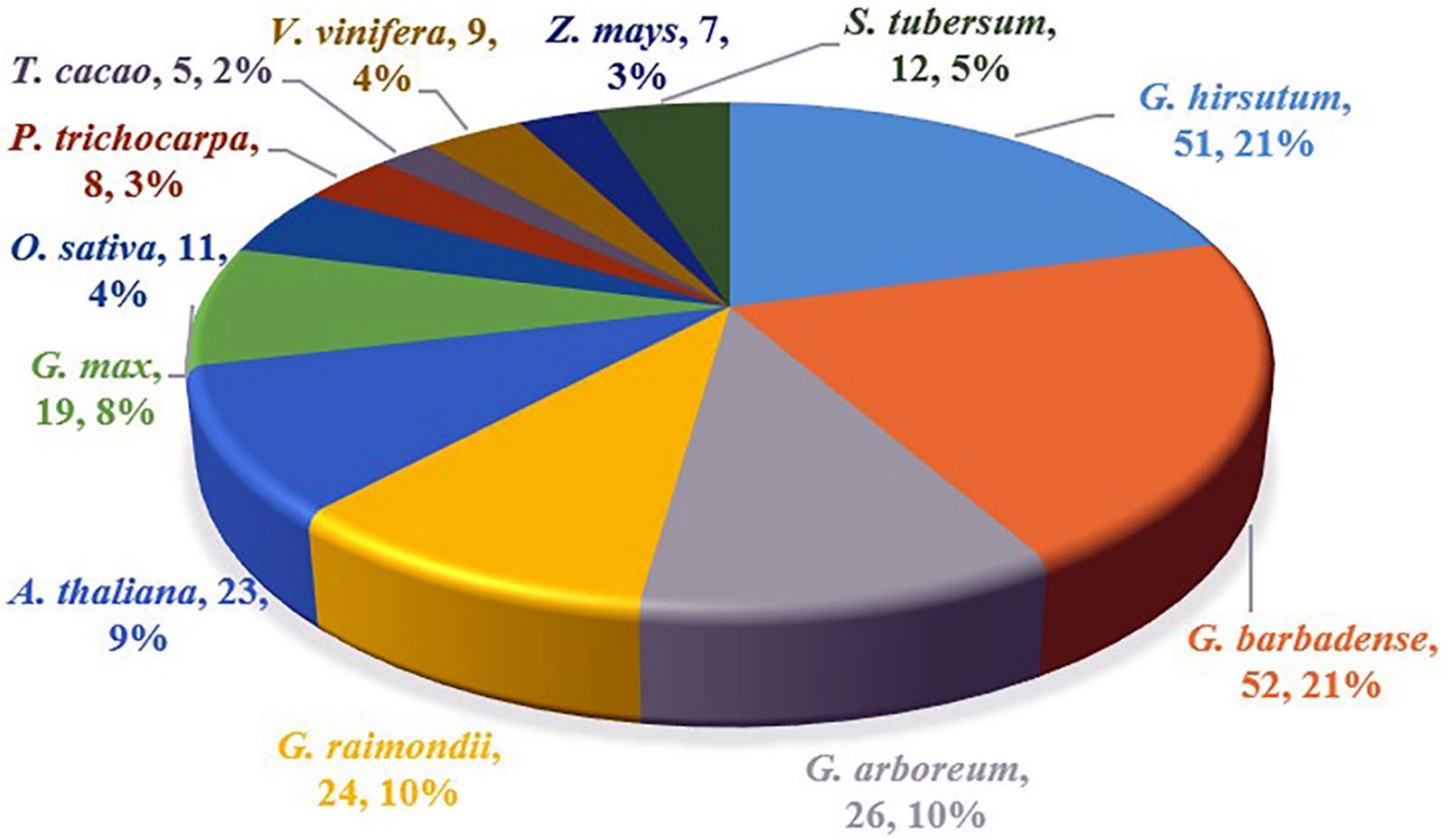
Distribution of NDA genes among twelve plant species

To identify the NDA gene family in cotton, a local blast on the proteome of the *G. hirsutum*, *G. barbadense*, *G. arboreum*, and *G. raimondii* was performed, and genome with the hidden Markov model (HMM) profile of PF07992 was used as a query condition. Redundant sequences were detected and deleted by manual, 51, 52, 26, 24 genes were identified in *G. hirsutum* (*GhNDAs*), *G. barbadense* (*GbNDAs*), *G. arboreum* (*GaNDAs*), and *G. raimondii* (*GrNDAs*), respectively (Fig. 1). Statistical analysis of the number of genes in four *Gossypium* species showed that the number of NDA genes in two tetraploid cotton species (*G. hirsutum* and *G. barbadense*) were almost double of diploid cotton species (*G. arboreum* and *G. raimondii*). To understand the genes of NDA family more conveniently, genes were renamed according to the position of genes on chromosomes. *GhNDA1*-*GhNDA51* was assigned for *G. hirsutum*, *GbNDA1*-*GbNDA52* for *G. barbadense*, *GaNDA1*-*GaNDA26* for *G. arboreum* and *GrNDA1*-*GrNDA24* for *G. raimondii*, other species were also renamed according to this rule (Additional file 2: Table S2). Subsequently, the physical properties of the *GhNDAs* genes were analyzed and predicted, including protein length, protein molecular weight (MWs), isoelectric point(pI), protein hydrophilicity and hydrophobicity analysis and subcellular location (Additional file 3: Table S3). For *G. hirsutum*, all of the 51 genes encoded proteins ranging from 357 (*GhNDA35*) to 2209 (*GhNDA*48) amino acids, with an average of 603.922 amino acids. The MWs varied from 38.742 (*GhNDA35*) kDa to 242.628 (*GhNDA*22) kDa with an average of 66.107 kDa and pI varied from 5.553 (*GhNDA36*) to 10.024 (*GhNDA35*) with a mean of 7.909. The values of the grand average of hydropathy were all negative, which proved that the proteins of NDA family were hydrophilic. The prediction of subcellular localization showed that there were 20 genes located in the mitochondria, 17 in cytoplasmic and 14 in chloroplast.

### Phylogenetic analysis of NDA genes

**Fig. 2:**
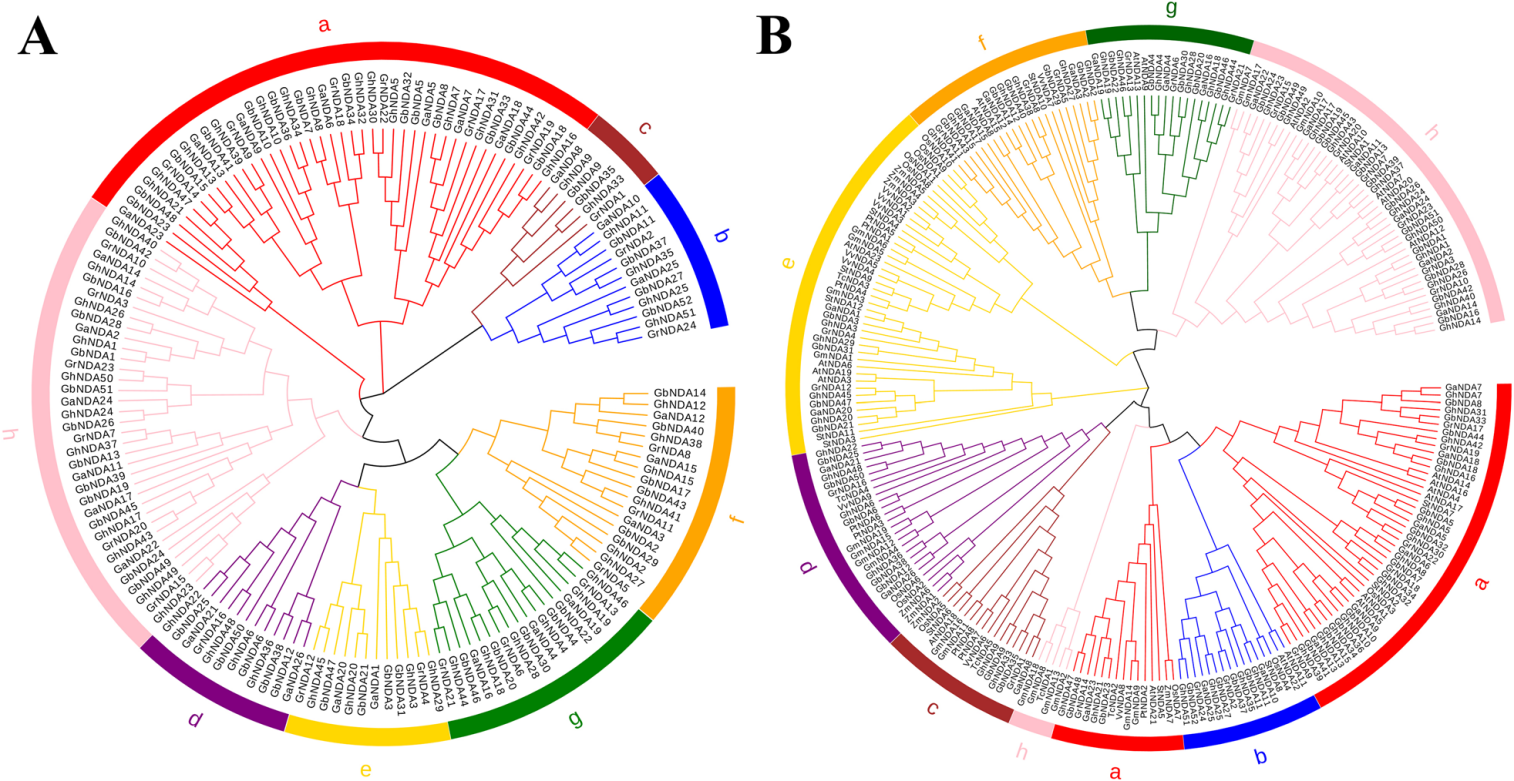
Phylogenetic trees of NDA genes in four *Gossypium* species and other eight species using MEGA 7 by the Neighbor-Joining (NJ) method. **A** Phylogenetic relationship of the 153 identified NDA genes from four *Gossypium* species, **B** Phylogenetic relationship of the 247 identified NDA genes from four *Gossypium* species and other eight species. The 8 major phylogenetic subgroups, designated as a to g, are marked with different colored backgrounds

To understand the evolutionary relationship of NDA genes among four *Gossypium* species and other plant species, multiple sequence alignment of 247 protein sequences (including 51 in *G. hirsutum*, 52 in *G. barbadense*, 26 in *G. arboreum*, 24 in *G. raimondii*, 23 in *Arabidopsis thaliana* (*A. thaliana*), 12 in *Solanum tuberosum* (*S. tubersum*), 19 in *Glycine max* (*G. max*), 11 in *Oryza sativa* (*O. sativa*), 8 in *Populus trichocarpa* (*P. trichocarpa*), 5 in *Theobroma cacao* (*T. cacao*), 9 in *Vitisvinifera Genoscope* (*V. vinifera*) and 7 in *Zea mays* (*Z. mays*)) was performed and a phylogenetic tree was constructed using MEGA 7 software based on the neighbor-joining (NJ) method. The phylogenetic tree was subsequently decorated using EvolView (https://www.evolgenius.info/evolview) (Fig. 2B). According to previous method of dividing the phylogenetic tree [22], we divided NDA genes into 8 clades based on the sequence similarity, tree topology and structural characteristics in each sequence. The clade a had the most extensive genes, of which 14 were *G. hirsutum*, 14 were *G. barbadense*, 7 were *G. arboreum*, and 6 were *G. raimondii*, 7 were *A. thaliana*, 3 were *S. tubersum*, 2 were *O. sativa*, 2 were *G. max*, 1 was *Z. mays*, 1 was *V. vinifera*, 1 was *T. cacao* and 1 was *P. trichocarpa*, respectively. All species had gene pairs derived from the same node, demonstrating that the NDA genes in all species had experienced gene duplication events that causing the expansion of the NDA gene family. The number of genes in *G. barbadense* and *G. hirsutum* was much higher than that of other species, indicating that the NDA gene family in two allotetraploid species showed a large-scale expansion during the evolution process. These results indicated that gene duplication was the main reason for the expansion of the NDA gene family in cotton.

In addition, to study the relationship between the common ancestors of diploid cotton (*G. arboreum* and *G. raimondii*) and allotetraploid cotton (*G. hirsutum* and *G. barbadense*), NJ tree of four *Gossypium* species was constructed (Fig. 2A). With reference to the classification of subgroups, we found that the NDA genes of the four *Gossypium* species were distributed in each subgroup, and each branch contained proteins from diploid and allotetraploid *Gossypium* species and the NDA genes in tetraploid cotton were almost twice as many as diploid cotton in each subgroup.

### Chromosomal location of four *Gossypium* species

**Fig. 3:**
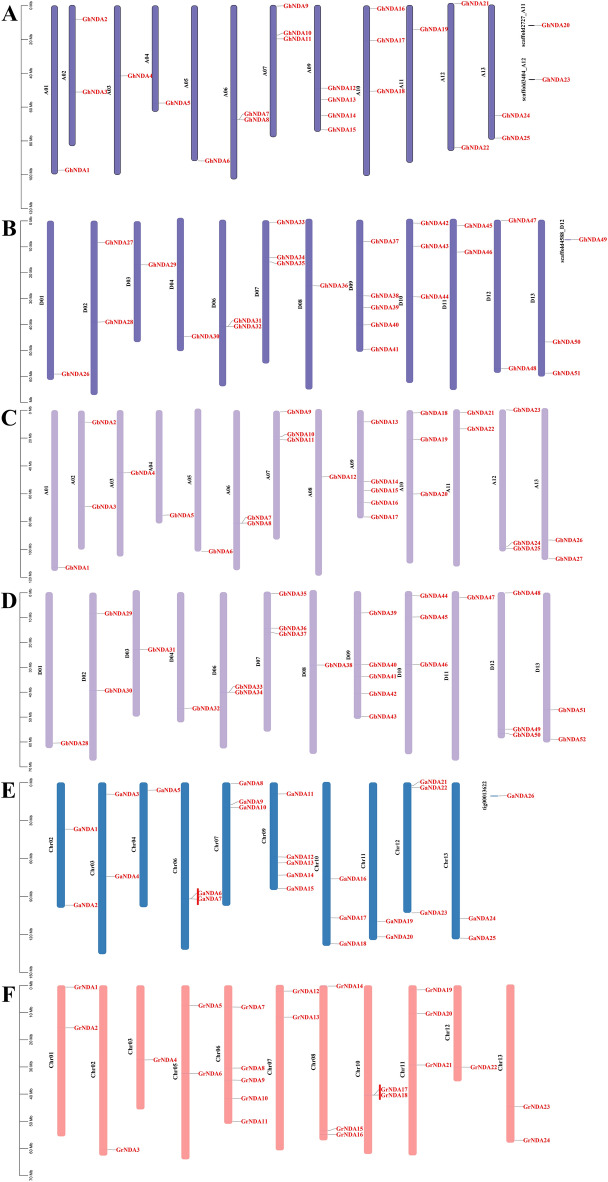
Chromosomal location of NDA genes from four *Gossypium* species. **A** Chromosomal location of NDA genes on chromosomes in *G. hirsutum* At sub-genome (GhAt), **B** Chromosomal location of NDA genes on chromosomes in *G. hirsutum* Dt sub-genome (GhDt), **C** Chromosomal location of NDA genes on chromosomes in *G. barbadense* At sub-genome (GbAt), **D** Chromosomal location of NDA genes on chromosomes in *G. barbadense* Dt sub-genome (GbDt), **E** Chromosomal location of NDA genes on chromosomes in *G. arboreum* (Ga), **F** Chromosomal location of NDA genes on chromosomes in *G. raimondii* (Gr) genes. The gene ID on the right side of each chromosome correspond to the approximate locations of each NDA gene. The scale of the genome size was given on the left. Red lines represented tandem gene duplications

To study the chromosomal distribution of NDA genes in four *Gossypium* species, the physical location of these genes on chromosomes was drew. 149 out of 153 NDA genes were distributed to their specific chromosomes, while the remaining only 4 NDA genes, *GhNDA20*, *GhNDA23*, *GhNDA49* and *GaNDA26*, were not located on any one chromosome because they were located on scaffold (Fig. 3). This result showed that the genetic evolution process of NDA genes was mature and stable. There was no *GhNDA* gene on Chr8 of *G. hirsutum* At sub-genome (GhAt), Chr5 of *G. hirsutum* Dt sub-genome (GhAt), Chr1, 5, 8 of *G. arboreum*, Chr4 of *G. raimondii*, Chr5 of *G. barbadense* (Fig. 3A, B, E, F), which may be related to the chromosome deletion of *G. hirsutum* or the translocation of large fragments during the evolution. The distribution of NDA genes on 13 chromosomes of different cotton species was uneven, and the number of each chromosome did not show a significant positive correlation with its length.

**Fig. 4:**
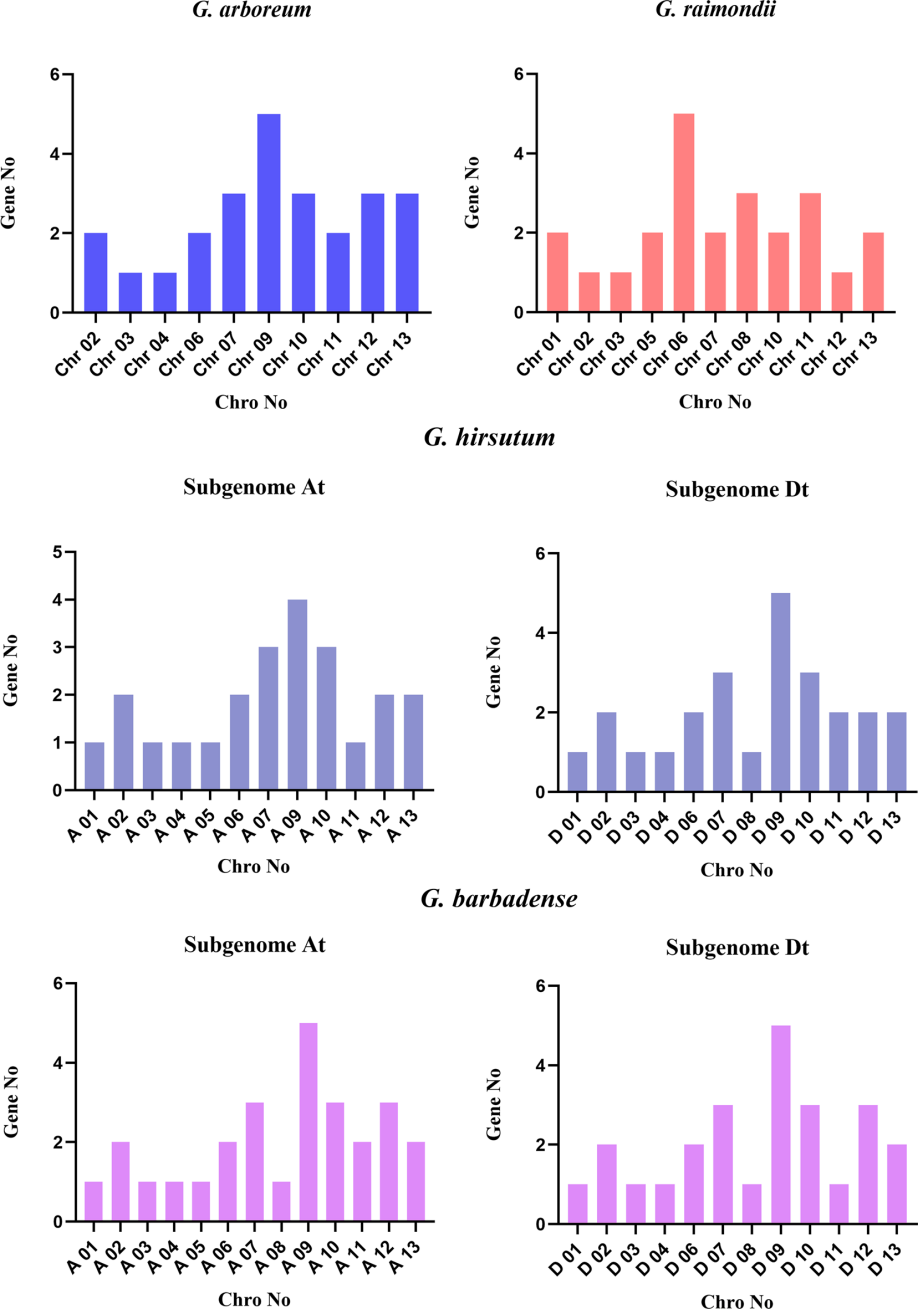
Number of NDA genes in each chromosome

Among 52 identified NDA genes of *G. hirsutum*, 3 genes were located on scaffold (Fig. 3A, B). Chr9 in GhAt had most NDA genes, with a total of 4 genes, and Chr9 in GhDt had most NDA genes, with a total of 5 genes (Fig. 4). In *G. barbadense*, Chr9 in GbAt and GbDt had most NDA genes, with a total of 5 genes, which was similar to the situation of *G. hirsutum* (Fig. 4). Among 26 identified NDA genes of *G. arboreum*, 25 genes were distributed on 13 chromosomes while one gene was found at scaffold (Fig. 3E). In *G. arboreum*, Chr9 had most NDA genes with a total of 5 genes while 5 genes in Chr6 in *G. raimondii* (Fig. 3E, F).

### Gene structure and motif composition analysis

**Fig. 5:**
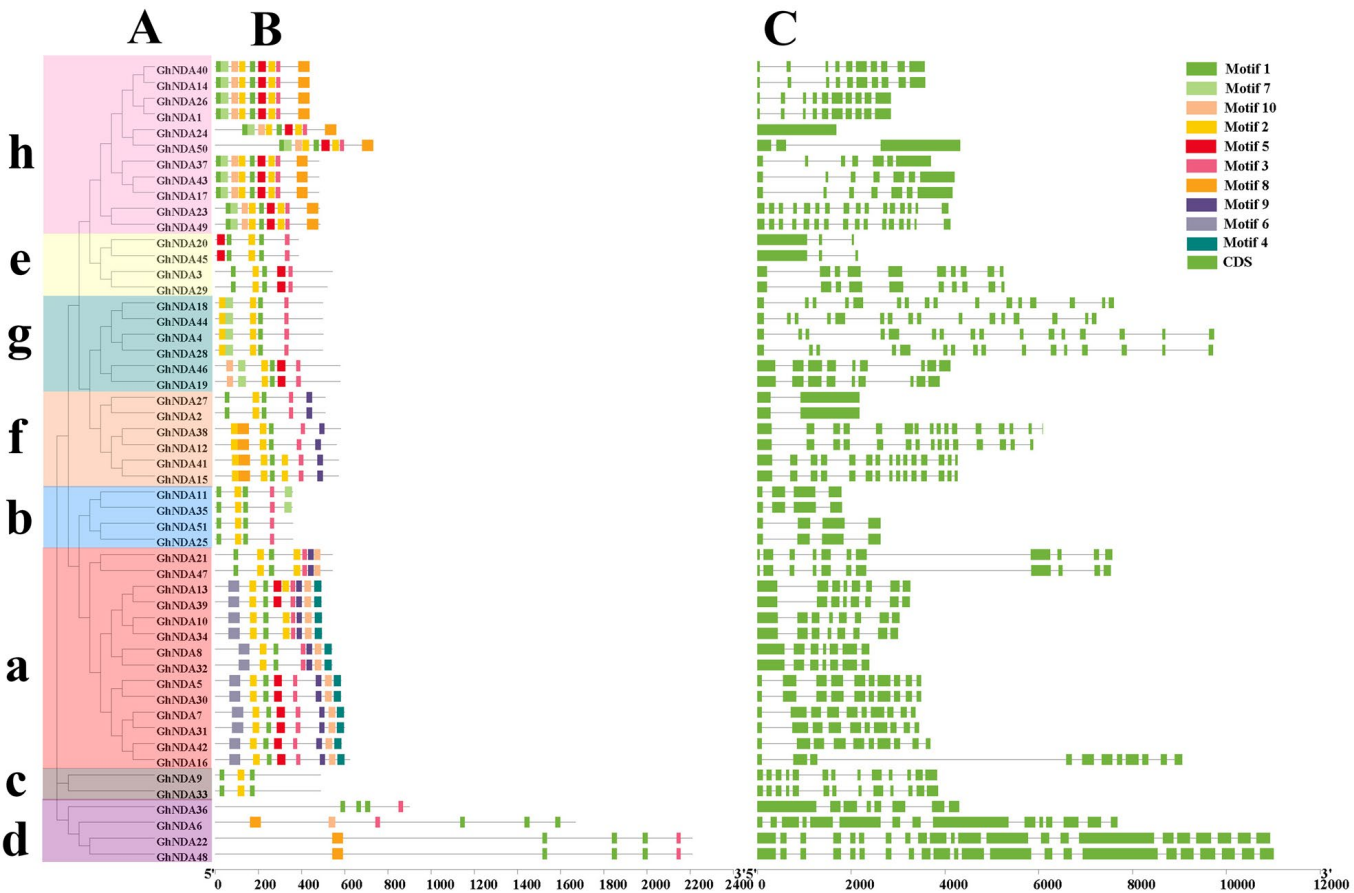
Phylogenetic analysis, conserved motifs, and gene structures of *GhNDA* genes. **A** Phylogenetic tree of *GhNDA* genes, the 8 major phylogenetic subgroups, designated as a to g, are marked with different colored backgrounds, **B** Conserved motifs of *GhNDA* genes, **C** Gene structures of *GhNDA* genes. Green boxes indicated exons; black lines indicated introns

To further examine the structural characteristics of the NDA genes in *G. hirsutum*, the conserved motifs of NDA proteins were predicted by using the online website MEME (http://meme-suite.org/). 10 conserved motifs were found (named motif 1 to motif 10) and the results were represented in schematic diagrams (Fig. 5B). The number of motifs was different in each protein, varying from 3 to 9. NDA members within the same clade were found to have a similar motif composition, which was conspicuous in class a, b, c, h, f. All the proteins belonged to the h and f subgroups contained motif 8, but the other clades did not had motif 8. Each clade had motif 1, and only clade a had the motif 4, while the other clade did not have, which may be the reason that clade a had obtained the special motif 4 through evolution or other clade had lacked specific conserved motifs during evolution. The different compositions of the motifs may represent the diversity of function. The clade a, b, c, h, f, h showed the similar motif arrangements, indicating that the protein was conserved within a specific subgroup. However, the functions of these conserved motifs remained to be further verified.

The diversity of gene structure was mainly influenced by the evolution of multigene families [23]. To further explore the diversity of NDA gene’s structure, the characteristics of exon-intron structures were analyzed. As can be seen from Fig. 5C, NDA genes of the same subfamilies had similar intron-exon arrangement, and different subfamilies displayed variation in exon-intron structures. The structures of NDA genes can be divided into two types, with intron less and multiple-exon. In *G. hirsutum*, the number of exons of all NDA genes varied from 1 to 22. Clade b had the least exon number (4), while Clade d had the most exon number (22). It was noting that one gene *GhNDA24* belonged to clade h had only one exon, no intron.

### Gene duplication and collinearity analysis

**Fig. 6:**
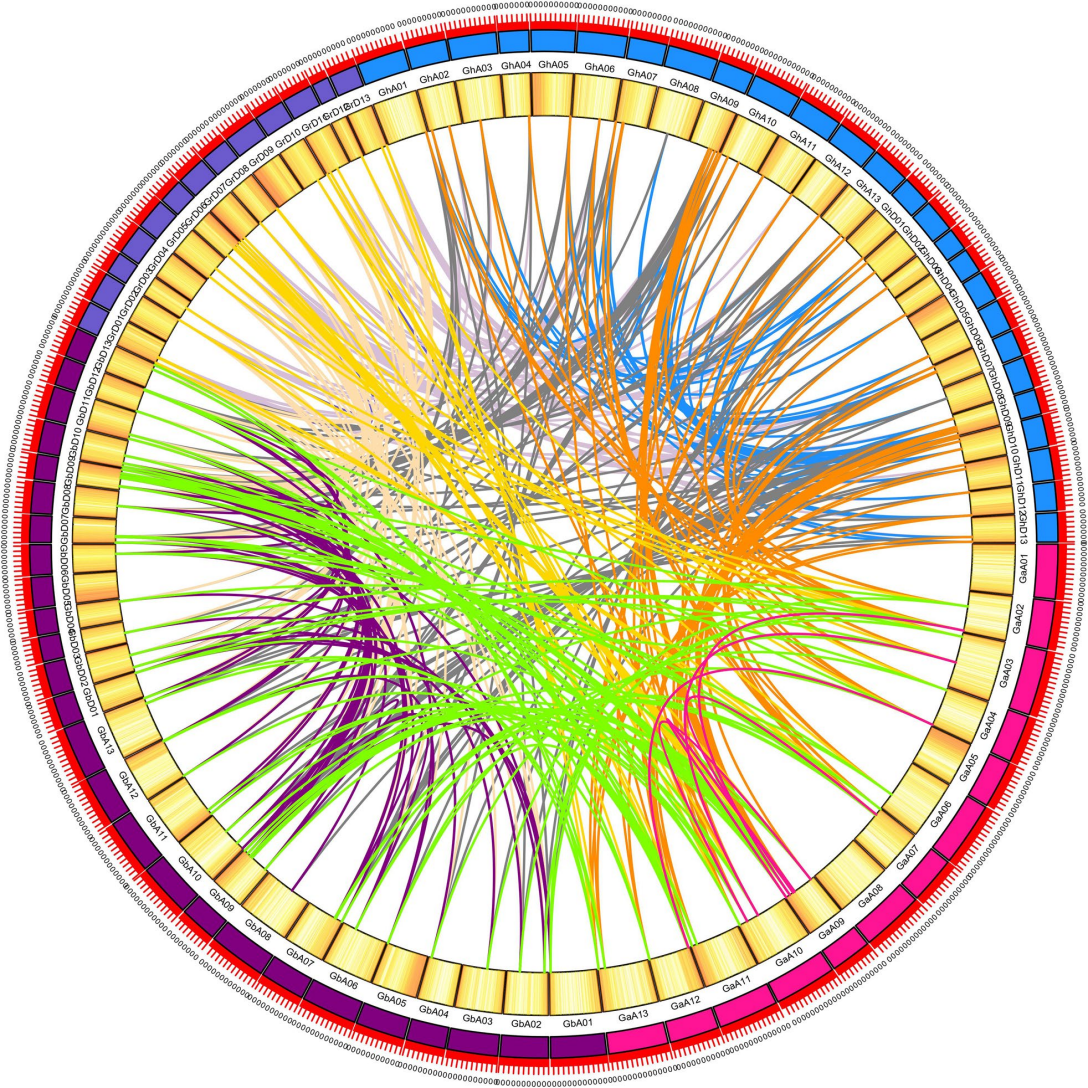
Syntenic relationship of duplicated NDA gene pairs from four *Gossypium* species (*G. hirsutum*, *G. barbadense*, *G. arboreum*, and *G. raimondii*). Chromosomal lines represented by various colors indicates the syntenic regions around the NDA genes, the duplication pairs were connected by lines. The heat map in the circle represented the density of genes on chromosome

In general, plants had a higher rate of gene replication than other eukaryotes. Whole genome duplication, segmental duplication, and tandem duplication were considered to be the main causes of expansion in plant gene family [24]. Segmental duplications in the genome region may result in the expansion of the gene family [25]. To investigate the evolutionary process of NDA genes, the gene duplication pattern of four *Gossypium* species was performed using MCScanX. In this study, a total of 361 gene pairs were identified as whole genome duplication, 2 gene pairs were the tandem duplication and 102 were the segmental duplication (Fig. 6). Among the 2 tandem repeat gene pairs, one was *GaNDA6*/*7*, the other was *GrNDA17*/*18* (Fig. 3E, F). The collinearity gene pairs between *G. hirsutum* and *G. barbadense* were the most among the 10 groups, with 80 gene pairs, while *G. raimondii* and *G. raimondii* had the least, with only 3 gene pairs, which was in line with the comparison of the number of diploid and tetraploid genes. From these results, we presumed that whole genome duplication and segmental duplication played an important role in the evolution of NDA gene family.

### Calculation of non-synonymous (*Ka*) to synonymous (Ks) substitution rates during evolution

**Fig. 7:**
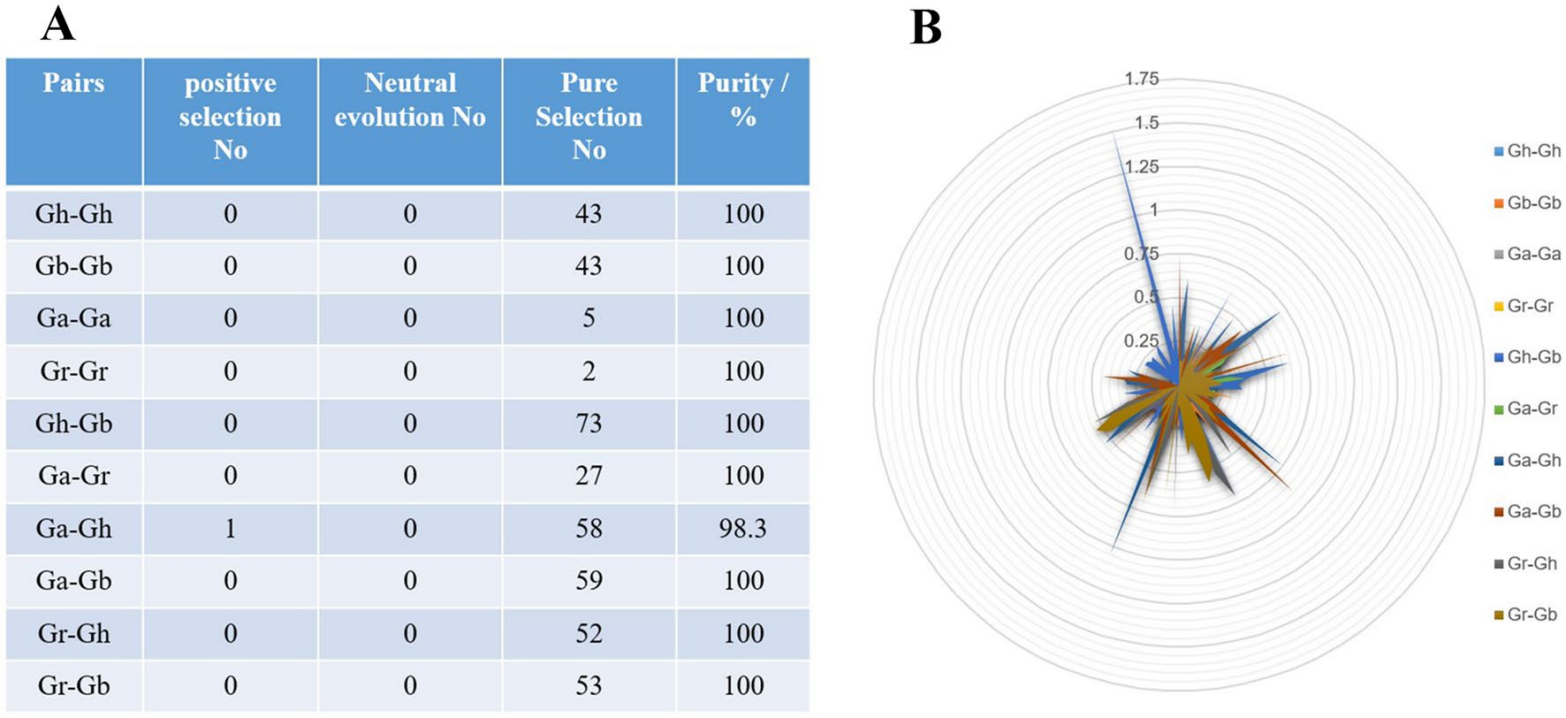
Analysis of non-synonymous (*Ka*) to synonymous (*Ks*) ratio. **A** Prediction of number of duplicated gene pairs involved in different combinations from four *Gossypium* species, **B** *Ka* and *Ks* divergence values for (Gh-Gh), (Gb-Gb), (Ga-Ga), (Gr–Gr), (Gh-Gb), (Ga-Gr), (Ga-Gh), (Ga-Gb), (Gr-Gh) and (Gr-Gb) are shown in circular chart. Different colors represent *Ka*/*Ks* gene pairs of 10 groups

To study the selection pressure of duplicated gene pairs in the evolutionary process, *Ka*, *Ks*, and *Ka*/*Ks* of 416 homologous gene pairs from 10 combinations of four *Gossypium* species were determined, including Ga-Ga, Ga-Gb, Ga-Gr, Gb-Gb, Gb-Gr, Gh-Ga, Gh-Gb, Gh-Gh, Gh-Gr and Gr-Gr (Additional file 4: Table S4). Among them, 2 (0.48%) duplicated gene pairs with *Ka*/*Ks* ratio > 1, which occurred in Gh-Ga and Gh-Gb, respectively, indicating that these genes may experience relatively rapid evolution and undergo the positive selection pressure. 414 (99.52%) duplicated gene pairs with *Ka*/*Ks* ratio < 1, exhibiting pure selection (Fig. 7).

### Gene ontology (GO) annotation analysis of *GhNDAs*

**Fig. 8:**
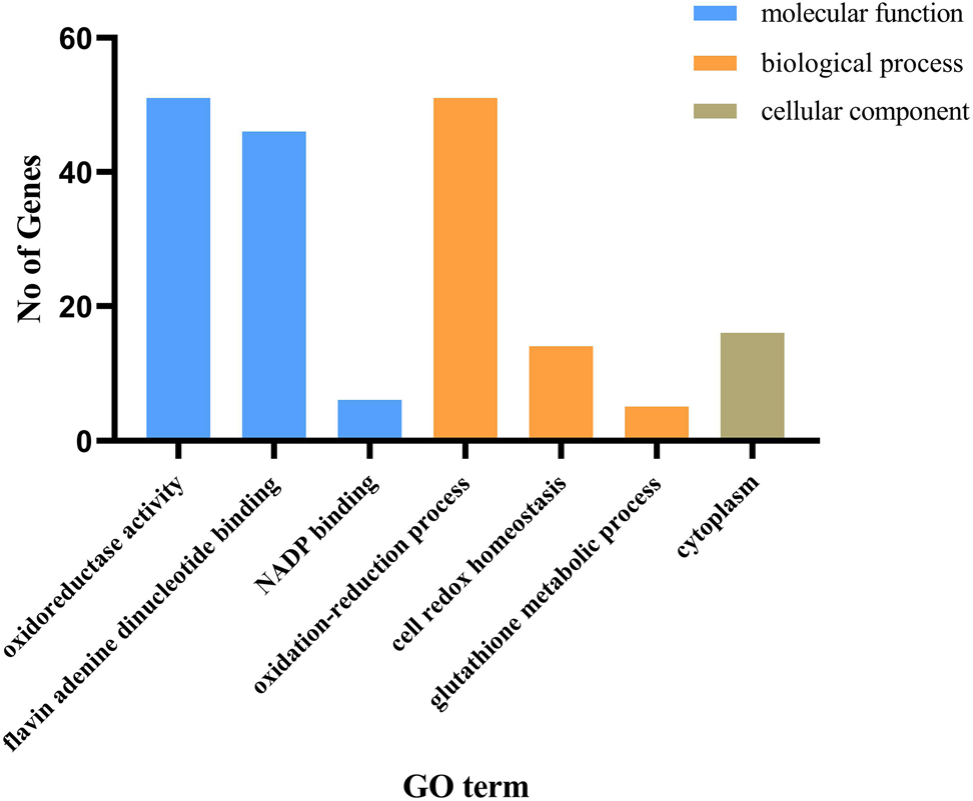
Gene ontology (GO) analysis of *GhNDA* genes

To further study the functions of *GhNDAs*, three major categories, including biological process, cellular component, and molecular function, were analyzed via CottonFGD (Fig. 8). According to GO analysis, we found that the molecular functions were mainly involved in oxidoreductase activity (51), flavin adenine dinucleotide binding (46) and NADP binding (6). Biological processes were mainly in the following three aspects, oxidation-reduction process (51), cell redox homeostasis (14) and glutathione metabolic process (5). The cellular components were mainly in cytoplasm (16).

### Analysis of promoters and expression profiles of *GhNDA* genes under alkaline stress

**Fig. 9:**
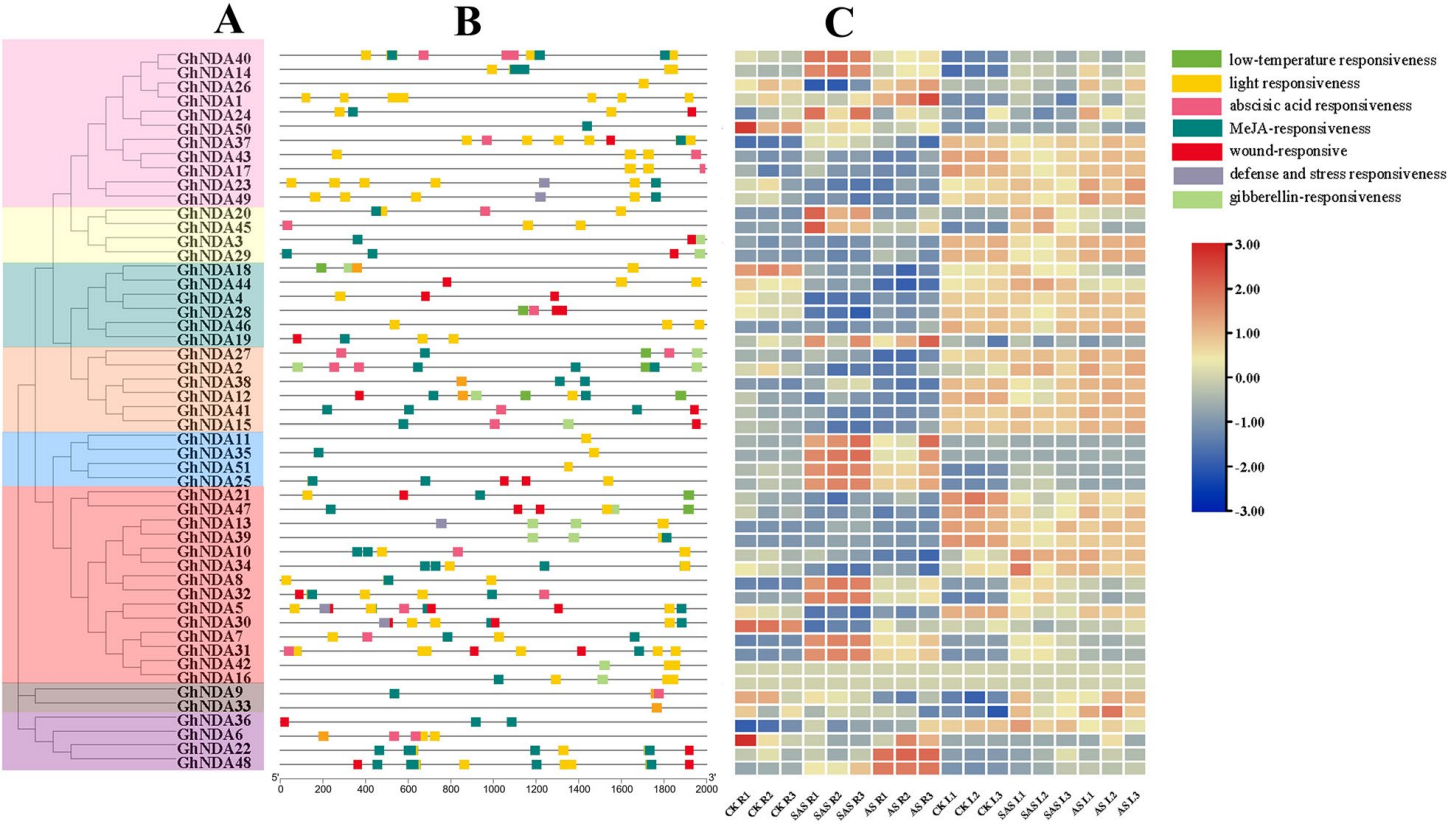
Analysis of promoters and differentially expressed genes of *GhNDA*. **A** Phylogenetic relationship of *GhNDA* genes, the 8 major phylogenetic subgroups, designated as a to g, are marked with different colored backgrounds, **B** *Cis*-acting elements in promoters of *GhNDA* genes, **C** Heatmap of DEGs of *GhNDA* genes under SAS and AS stress. SAS: 125 mM NaHCO_3_ (pH = 8.5); AS: pH = 8.5 NaOH

*Cis*-acting elements played vital roles in the response to abiotic stress [26]. Some plant hormones such as ethylene (ET), abscisic acid (ABA), methyl jasmonate (MeJA) and gibberellin (GA) were necessary for plants to adapt to abiotic stress, and some transcription factors could combine with hormone-related *cis*-acting elements to regulate the expression of genes [27, 28]. To identify putative *cis*-acting elements in the NDA genes, the region of 2000 bp upstream of the start codon of the NDA genes was selected, and the promoter region related to stress and plant hormones was extracted. In this study, most *GhNDA* genes in *G. hirsutum* contained *cis*-acting elements related to plant hormones (ABA, MeJA, GA,) and various stresses (low temperature, light and wound stress) (Fig. 9B), the *cis*-acting elements of NDA genes in the same subfamily were different despite had similar motifs, and therefore their functions may differ. Most of NDA genes contained *cis*-acting elements related to plant hormones and various stresses. *GhNDA5* had 11 *cis*-acting elements (3 were light responsiveness, 1 were abscisic acid responsiveness, 3 were MeJA responsiveness, 3 were wound responsiveness), whereas *GhNDA33* and *GhNDA11* had only one *cis*-acting element (light responsiveness). Almost all *GhNDA* genes were involved in MeJA *cis*-responsive elements except clade g.

It was found that gene expression levels were highly correlated with gene function [29]. We detected the expression pattern of *GhNDA* genes under different stresses (AS, SAS) by analyzing RNA-seq data. By analyzing the heat map, we found that only four genes, *GhNDA8*, *GhNDA14*, *GhNDA32* and *GhNDA40*, only responded to SAS stress, *GhNDA1*, *GhNDA22*, *GhNDA26* and *GhNDA48* only responded to AS stress, *GhNDA11*, *GhNDA19*, *GhNDA25*, *GhNDA35* and *GhNDA51* responded to both AS and SAS stress in roots (Fig. 9C). In addition, we found that most of the gene expression levels in leaves were higher than those in roots (Fig. 9C). The expression patterns of NDA genes in roots and leaves were different, which required us to use qRT-PCR to further verify the differences in gene expression.

### Expression analysis of *GhNDA* genes under different alkaline stress

**Fig. 10:**
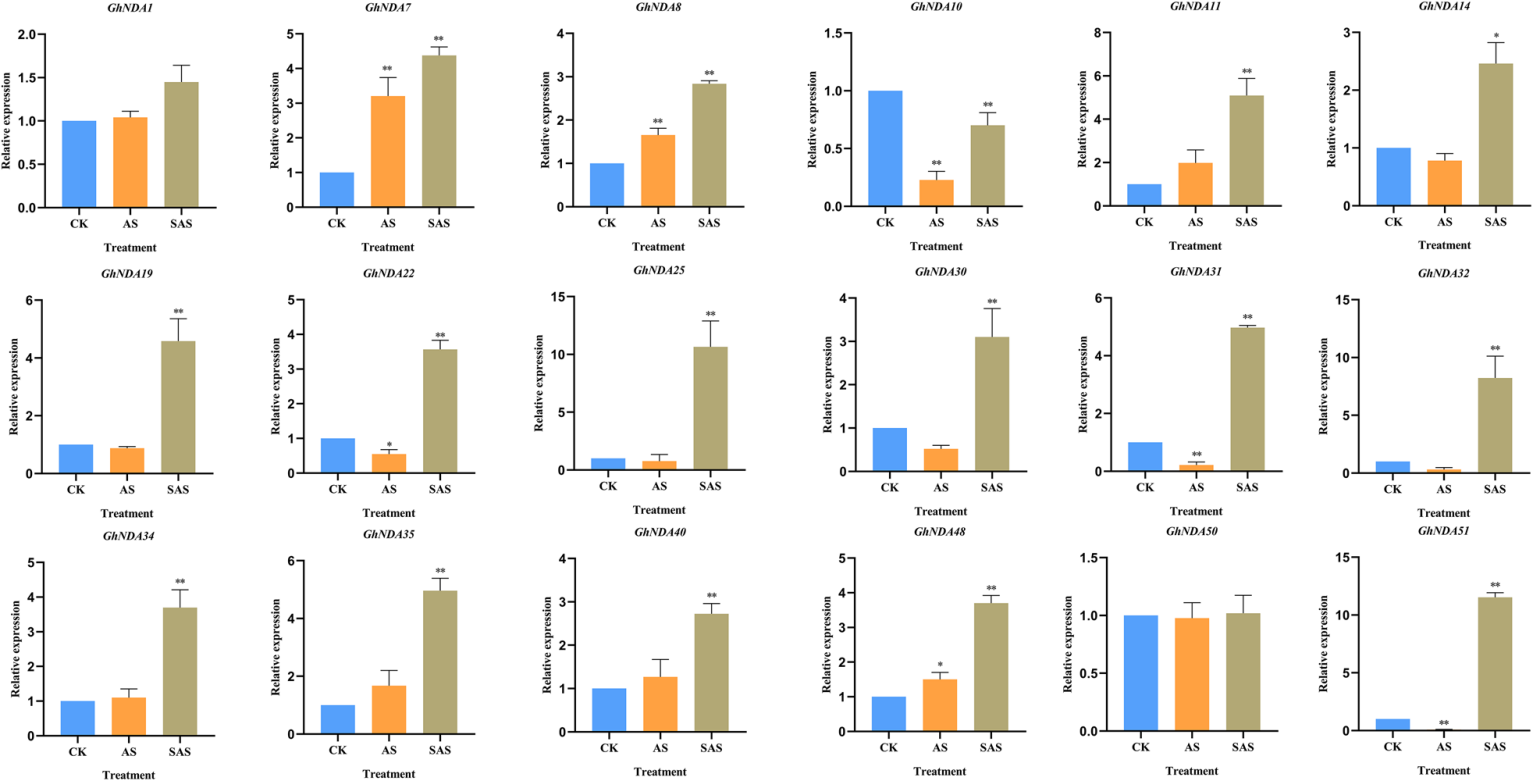
Expression analysis of *GhNDA* genes in response to different alkaline stress in roots using qRT-PCR assays. Cotton seedlings were treated with 125 mM NaHCO_3_ for 12 h. The mean values were from three independent biological replicates. Statistical analyses were performed by Student’s t-test (*P < 0.05 and **P < 0.01)

**Fig. 11:**
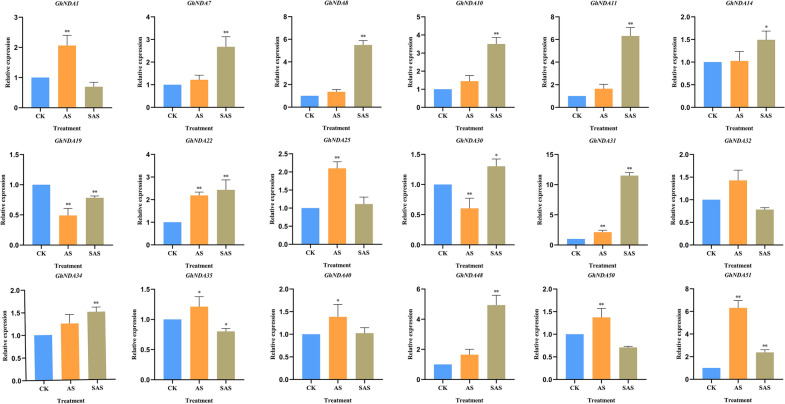
Expression analysis of *GhNDA* genes in response to different alkaline stress in leaves using qRT-PCR assays. Cotton seedlings were treated with 125 mM NaHCO_3_ for 12 h. The mean values were from three independent biological replicates. Statistical analyses were performed by Student’s t-test (*P < 0.05 and **P < 0.01)

After being exposed to AS and SAS stress, qRT-PCR assays were conducted to explore the expression patterns of *GhNDA* genes in roots and leaves (Figs. 10, 11). 18 genes were picked up randomly from the NDA genes actively responding to SAS and AS stress for further analysis through qRT-PCR. We found that the *GhNDA* genes all responded to alkaline stress, although they showed different up-regulation relationships. In addition, *GhNDA7*, *GhNDA8*, *GhNDA48* showed extremely significant up-regulation under AS stress in roots, *GhNDA1*, *GhNDA22*, *GhNDA25*, *GhNDA35*, *GhNDA40*, *GhNDA50*, *GhNDA51* showed extremely significant up-regulation under AS stress in leaves. Almost all genes were responsive to SAS stress in roots except *GhNDA1* and *GhNDA50*. At the same time, gene expression in leaves also showed a similar trend to that in roots, except *GhNDA1*, *GhNDA25*, *GhNDA32*, *GhNDA40* and *GhNDA50*. Therefore, we believed that most *GhNDA* genes responded to both AS and SAS stress.

### Cotton plants with *GhNDA* gene silenced by VIGS were sensitive to NaHCO_3_ alkaline stress

**Fig. 12:**
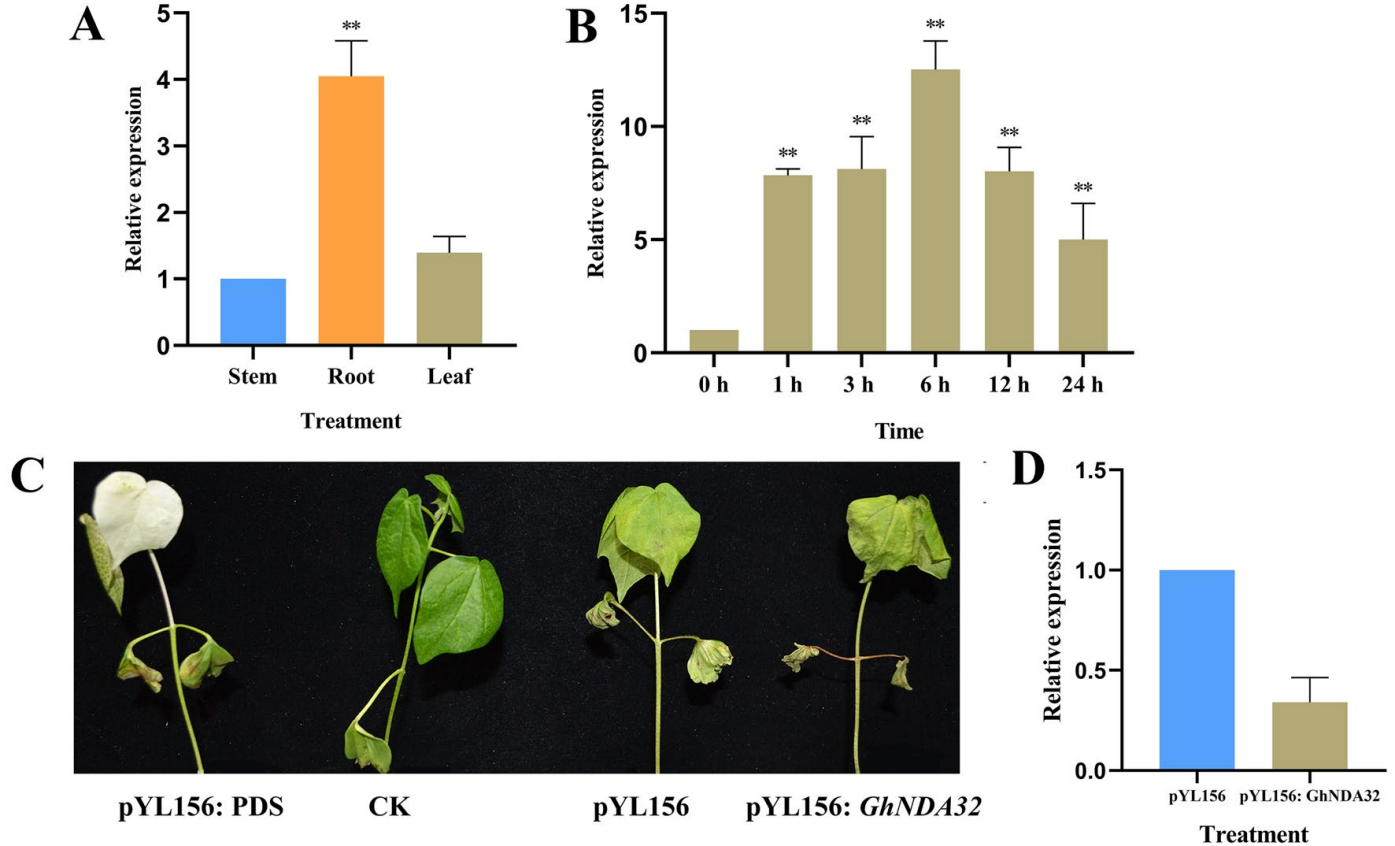
The phenotype of cotton leaves after virus infection and expression analysis of *GhNDA32* under NaHCO_3_ stress. **A** Analysis of relative expression of *GhNDA32* in roots, stems and leaves, **B** The relative expression of *GhNDA32* in different time periods under SAS stress, **C** Phenotype of cotton leaves after virus infection, **D** qRT-PCR for *GhNDA32* under SAS stress. CK: No infection, SAS: 125 mM NaHCO_3_

To verify whether the *GhNDA* genes responded to alkaline stress, a high-expressed gene *GhD06G1340.1* (*GhNDA32*) from transcriptome data was selected for further study. First, we tested the tissue specificity of the *GhNDA32* gene, and the results showed that the relative expression in roots was the highest, followed by leaves and stems (Fig. 12A). Then we analyzed the relative expression of *GhNDA32* gene at different time periods treated with alkaline stress (125 mM NaHCO_3_) and it showed a trend of first increasing and then decreasing, while reached the peak at 6 h. After that, a VIGS silencing vector pYL156: *GhNDA32* was constructed, when the seedlings carrying pYL156: PDS appeared albino phenotype, qRT-PCR was used to detect the expressions of *GhNDA32* gene in the pYL156 plants and pYL156: *GhNDA32* plants, the results showed that the expression level of *GhNDA32* in the pYL156: *GhNDA32* was significantly lower than that of the control pYL156 (Fig. 12D), indicating that gene silencing was successful. After treatment with 125 mM NaHCO_3_, the leaves of the seedlings all appear wilting, and we found that the pYL156: *GhNDA32* cotton seedlings wilted more severely than the pYL156 (Fig. 12C). Therefore, *GhNDA32* was also involved in the response to alkaline stress.

### Interaction network of GhNDA32 protein

**Fig. 13:**
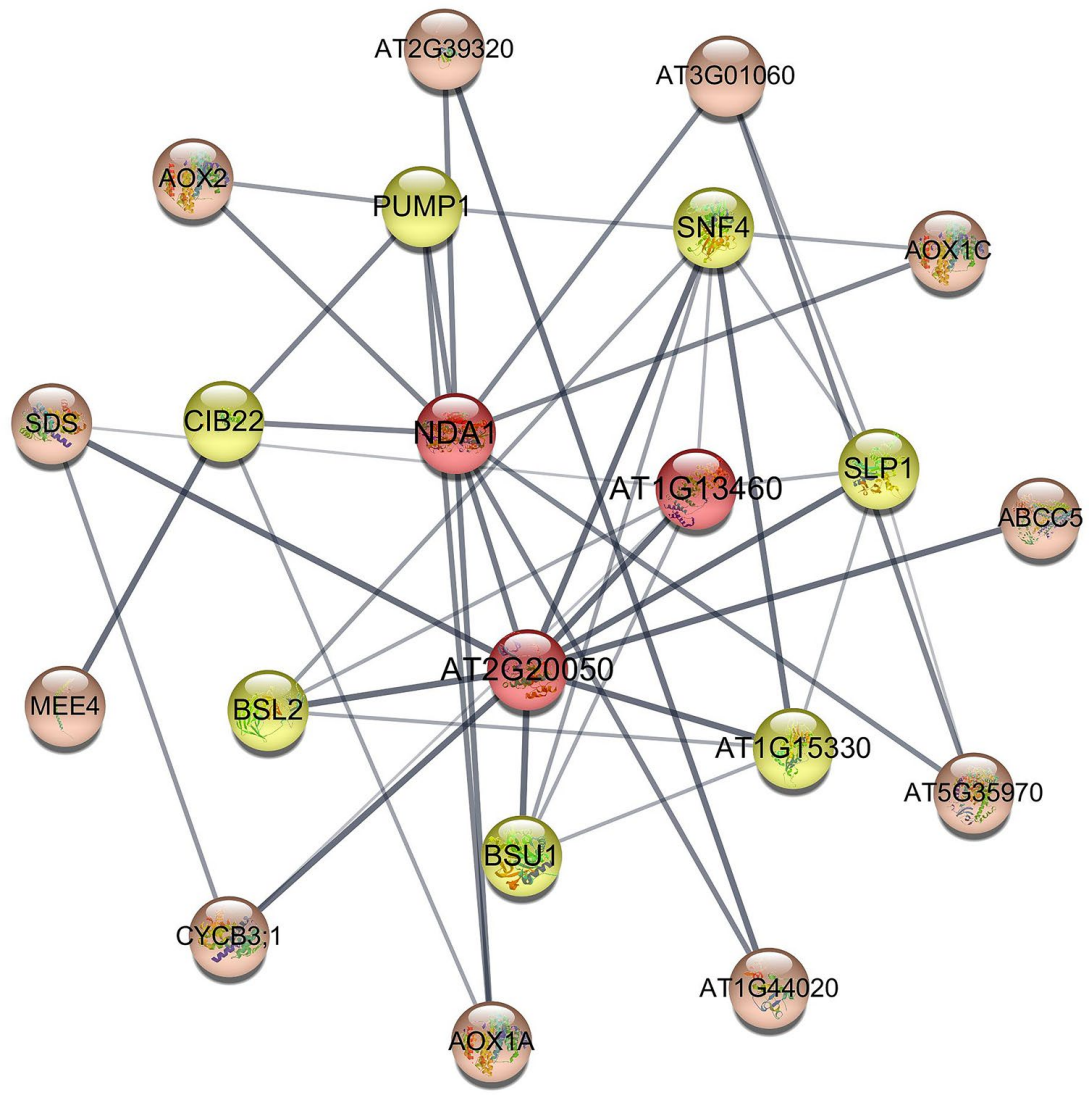
Interaction network of GhNDA32 protein. The NDA1 represented the protein AtNDA1 corresponding to the protein in *Arabidopsis* with the highest homology to GhNDA32

According to the homologous gene in *Arabidopsis*, we used STRING database (https://string-db.org/) to construct an interaction network to analyze the function of GhNDA32 protein (Fig. 13). From the Fig. 9, we could conclude that NDA1 protein of *Arabidopsis*, which was homologous to GhNDA32 protein of *G. hirsutum*, could interact with AOX1A, AOX1C, AOX2, PUMP1, and CIB22 proteins. By analyzing KEGG pathway of GhNDA32 protein from the transcriptome data, we found that GhNDA32 protein was mainly involved in oxidative phosphorylation (ko00190), so we speculated that GhNDA32 protein interacted with AOX1A, AOX1C, AOX2, PUMP1 and CIB22 proteins, which could remove excess reducing energy and balance the redox poise of the cell, thereby improving the resistance of cotton seedlings to oxidative stress.

## Discussion

Cotton is a major cash crop in the world and is also a powerful model for studying genome polyploidization in plants [30]. With the completion of *de*-*novo*-assembled genome of *G. hirsutum*, *G. barbadense* [31] and *G. arboreum* [32], this undoubtedly provides us with an important basis for the whole genome analysis of cotton. In the past several years, studies found that the ND family was mainly involved in the response to abiotic stress, such as drought, elevated light treatments and oxidative stress [10, 33], and NDA family genes were mainly in the response to cold stress and photorespiration [15, 34]. However, no systematic study of NDA genes is conducted in cotton, especially under alkaline stress. In this work, we undertook a comprehensive analysis of cotton NDA family and their involvement in alkaline stress.

In this study, 51, 52, 26 and 24 candidate NDA genes were systematically identified in *G. hirsutum*, *G. barbadense*, *G. arboreum* and *G. raimondii*, respectively. The NDA genes of *G. hirsutum* and *G. barbadense* were almost twice the number of *G. arboretum* and *G. raimondii*, which may be the reason that allotetraploid cotton was formed by inter-genomic hybridization of At-genome diploids and Dt-genome diploids, and followed by chromosome doubling [35]. In *G. hirsutum* and *G. barbadense*, the number of homologous NDA genes in At and Dt subgenome was identical, so we could infer that the translocations and reverse transcript insertion rarely occurred in the generation of cotton NDA gene family. The distribution of NDA varied greatly among selected plant species, such as 5 in *T. cacao*, compared with 51 in *G. hirsutum*, indicating that NDA genes underwent large scale expansion in higher plants. By analyzing the physical properties of *GhNDAs*, we found that the average MWs of *GhNDAs* were 66.107 kDa, which were consistent with the situation of the potato and *Arabidopsis* with the molecular weight of 55–57 kDa [11].

According to phylogenetic characteristics and gene structure, the NDA genes can be divided into 8 clades in four *Gossypium* species (Fig. 2A), and clade a and clade h had the largest number of NDA genes with 59 and 35, respectively. By analyzing the motif composition of NDA genes in *G. hirsutum*, it was worth noting that clade a and clade h contained the largest number of motifs, clade a had a specific motif 4 and none of the other clades, while clade h had motif 7 but clade a did not. Gene structure analysis showed that exon number of NDA genes varied greatly from 1 to 22, which might be due to the directional evolution in the function and structure of NDA genes in the process of evolution. There was a strong connection between the intron-exon structure, conserved motifs and phylogenetic tree analysis of the NDA family in *G. hirsutum*. Therefore, we speculated that the differences in gene structure and motifs led to different branches of the NDA family, and conserved motifs could also reflect the relations of different subgroups. For example, both clade a and f had motif 9, while other clades did not, which may be related that they had acquired some important functions during evolution, and this might be used as an important basis for identifying these two subgroups.

When analyzing the chromosomal location of *G. hirsutum* and *G. barbadense*, we found that there were 25 NDA genes in GhAt and 27 genes in GbAt. Interestingly, there was no gene distribution on chromosome 8 of GhAt, which proved that chromosome changes may occur a translocation or the gene on the chromosome 8 was lost in the process of evolution. In addition, there were two genes on chromosome 11 of GhAt, but only one gene on chromosome 11 of GbAt, which proved that *G. hirsutum* had lost a gene or *G. barbadense* gained a new gene through evolution. Similarly, there were 26 NDA genes in GhDt and 25 genes in GbDt, and the only difference of chromosomal location of GhDt and GbDt was that the GbDt lacked a gene on chromosome 11 than the GhDt, and the same was that they all did not have the chromosome 5. Our results showed the uneven distribution of NDA genes among all the four *Gossypium* species, which might be due to the addition or gene loss of NDA genes through tandem, segmental, or whole genome duplication events during evolution.

Studies had found that gene duplication was the most commonly evaluated mechanism for the divergence of gene family [36]. To reveal the expansion mechanism of the NDA gene family, duplicated gene pairs of four *Gossypium* species were identified by MCScanX, we found that only two tandem duplication events existed in both *G. arboretum* and *G. raimondii*, *GaNDA6*/*7* and *GrNDA17*/*18*, while the rest of duplicated gene pairs was identified as segmental duplication and whole genome duplication. This results revealed that low-tandem and high-segmental duplications were existed in the NDA gene family and segmental duplication and whole genome duplication played a predominant driving force in the evolution of NDA gene family, and it was consistent with the previous research [37].

To elucidate the differences after gene duplication, the ratio of non-synonymous (*Ka*) to synonymous (*Ks*) of four *Gossypium* species was calculated. In general, *Ka*/*Ks* < 1 was considered as a purification selection, indicating that natural selection eliminated harmful mutations and kept the protein unchanged, *Ka*/*Ks* > 1 was considered as positive selection, indicating natural selection changed the protein, the mutation site was quickly fixed in the population, and the evolution of the gene was accelerated, *Ka*/*Ks* = 1 was considered as neutral selection, indicating that natural selection did not affect mutation [38]. We found the *Ka*/*Ks* of most duplicated gene pairs in the four *Gossypium* species was less than 1, indicating that the four *Gossypium* species had undergone strong purification selection that occurred after tandem, segmental, and whole genome duplication. This was consistent with recent research that most cotton *GRX* gene family evolved through purifying selection pressure [39]. However, the selection pressure of most gene pairs among Gb-Gr, Gh-Gr, Gh-Ga and Gb-Ga were generally at 0–0.49, indicating that NDA genes tended to be conservative in evolution. Only the *Ka*/*Ks* value of Gh-Ga was greater than 1, suggesting that a certain member of *GaNDA* gene family members were evolved into some *GhNDA* gene family members through the environmental selection pressure, but whether they brought harmful traits or beneficial traits remains to be further studied.

Usually, *cis*-acting elements played an important role when plants were subjected to abiotic stress, and transcription factors (TFs) could bind to *cis*-acting elements of the promoter to regulate the transcription process and ultimately lead to the expression of genes [26]. In this study, most of the NDA family genes responded to light, which was consistent with the previous light-induced expression of *NDA1* [15]. Light was a vital regulator of gene expression in plants, altering the transcription of thousands of genes [40]. However, the vast majority of genes related to light response focused on photosynthesis-associated nuclear genes, little was known about the effects of light on mitochondria and the respiratory chain [41, 42]. In this study, most of the NDA family genes responded to light, therefore, we inferred that NDA family genes in upland cotton actively responded to light and the light could induce gene expression in mitochondria. Electron transport chains are known to produce ROS, which expose cells to oxidative stress. According to our GO annotation analysis (Fig. 8), most biological processes were concentrated in the oxidation-reduction process, which indicated that the NDA family genes were involved in the oxidation-reduction balance in cells to cope with alkaline stress. As cotton NDA genes are predicted to be localized in multiple subcellular compartments including the cytosol, chloroplasts, and mitochondria (Additional file 3: Table S3), it seems logical that its oxidation-reduction system should also be present in these compartments. In addition, a large number of hormone responsive elements (MeJA, ABA) in the promoters of *GhNDAs*, indicating that plant hormones probably involved in the regulation of *GhNDAs* in the upstream.

The expression pattern of genes was often used as an indicator of their functions. Therefore, RNA-Seq data was used to detect the possible function of genes in the NDA gene family by expression levels. Heat map analysis showed that most genes could be affected by SAS and AS stress (Fig. 9C), suggesting that NDA family genes play important roles in the response to alkaline stresses. The expression pattern of genes in different clades was different, but all the genes in the clade b were involved in the response of SAS and AS stress, and the *cis*-acting elements in the clade b were all involved in light-responsiveness, which demonstrated that *GhNDAs* in clade b had the function in response to light stress. In addition, some genes actively responded to SAS stress, such as *GhNDA40*, *GhNDA14*, *GhNDA20*, *GhNDA45*, *GhNDA11*, *GhNDA35*, *GhNDA51*, *GhNDA25*, *GhNDA32* and *GhNDA5*, while *GhNDA1*, *GhNDA14*, *GhNDA22* and *GhNDA48* actively responded to AS stress. Usually, NAD(P)H dehydrogenases generally may be up-regulated under stress, and the results of qRT-PCR analysis showed that most *GhNDA* genes are up-regulated under AS and SAS stress (Figs. 10, 11). *GhNDA25* and *GhNDA32* had the highest relative expression levels in roots while not in leaves, showing that these two genes may perform specific functions or participate in important signal pathways in the roots. In summary, the differential expression of several *GhNDA* genes indicated that they played an important role in alkaline stress response, which provided us with important candidate genes for studying alkaline stress in cotton.

Under alkaline stress, *GhNDA32* gene expression was induced in cotton. However, alkaline stress can also destroy the oxidation-reduction balance of plants, resulting in cotton leaves wilted with water loss. In our study, the wilting degree of VIGS plants was higher than that of non-VIGS plants, indicating that the alkaline tolerance of cotton decreased after *GhNDA32* gene was silenced. According to GO annotation, the biological process *GhNDA32* gene participated in is oxidation-reduction process, with molecular function as oxidoreductase activity. Combining the results of GO annotation, VIGS and the interaction network diagram, we could infer that *GhNDA32* gene might interact with AOX1A, AOX1C, AOX2, PUMP1 and CIB22 protein to regulate the activity of NADH dehydrogenase to regulate the redox reaction in the cotton, thereby eliminating the ROS produced in the body and maintaining cotton from the harm of oxidative stress.

Overall, this study will contribute to further understanding of the biological and molecular functions of cotton NDA and their antioxidant effects under alkaline stress. Our analysis provides a framework for further research into the function of this important gene family. The results of this study provide useful information for studying the effect of NDA genes in upland cotton under alkaline stress. These findings not only provide useful information for studying the effects of the NDA genes in *G. hirsutum* under alkaline stress but also provide valuable information for potential candidate genes related to plant growth and development and adversity stress.

## Conclusions

In this study, phylogenetic relationship, gene structure, chromosomal distribution, collinearity analysis as well as *cis*-acting elements were conducted, which largely enriched our knowledge of the cotton NDA gene family. In addition, most *GhNDAs* contained *cis*-acting elements like light responsiveness. Expression patterns and functional characterization indicated *GhNDAs* participated in response to alkaline stress, especially NaHCO_3_ stress. Taken together, all these results were of great significance for the future research on the molecular mechanism of NDA gene family in responding to abiotic stresses.

## Materials and methods

### Databases

Genome sequences of four *Gossypium* species (*G. hirsutum*, NAU; *G. barbadense*, HAU; *G. arboreum*, CRI; and *G. raimondii*, JGI) were used to identify the gene family. Genomic sequences, coding sequences of all of the four species were downloaded from Cotton Functional Genomic Database (CottonFGD) (http://www.cottonfgd.org/) [43]. Protein sequences of other species like *A. thaliana* (TAIR 10), *T*. *cacao* (version 10), *S. tuberosum* (version 4.03), *O*. *sativa* (version 7), *P*. *trichocarpa* (version 3.0), *V*. *vinifera Genoscope* (version 12), *G*. *max* (version 10), *Z*. *mays* (version 10) were obtained from Phytozome v12.1 (https://phytozome.jgi.doe.gov/pz/portal.html).

### Identification of NDA family members

To identify the members of the *GhNDA* gene family, the protein sequence and genome annotation were downloaded from CottonFGD. The Hidden Markov Model (HMM) of PF07992 was downloaded from the Pfam website (https://pfam.xfam.org/). Local blast was used to obtain the protein sequence of PF07992, NCBI Batch Web CD-Search Tool (https://www.ncbi.nlm.nih.gov/Structure/bwrpsb/bwrpsb.cgi) was used to further screen the genes [44]. CottonFGD was used to obtain some other features of *GhNDA* genes like protein length, molecular weights (MWs), isoelectric points (pIs), grand average of hydropathy. Subcellular location of GhNDA proteins were predicted using several websites, such as WOLF-PSORT (https://wolfpsort.hgc.jp/)  and CELLO v.2.5 (http://cello.life.nctu.edu.tw/) [45].

### Phylogenetic analysis and sequences alignments

The full-length amino acid sequences of NDA genes for twelve plant species including *G. hirsutum, G. barbadense, G. arboreum, and G. raimondii* (downloaded from CottonFGD), *A. thaliana* (TAIR 10), *T. cacao* (version 10), *S. tuberosum* (version 4.03), *O. sativa* (version 7), *P. trichocarpa* (version 3.0), *V. vinifera* (version 12), *G. max* (version 10), *Z. mays* (version 10) (downloaded from Phytozome v12.1) were provided in MEGA7 software using ClustalW program for multiple sequence alignment. Subsequently, MEGA 7 software [46] was used to construct phylogenetic tree using neighbor joining (NJ) method with default parameters.

### Chromosomal locations of NDA genes from four *Gossypium* species

The diagrams of the chromosomal locations from four *Gossypium* species including *G. hirsutum*, *G. barbadense*, *G. arboreum* and *G. raimondii* were drawn with the help of TB Tools software [47] by using the Generic Feature Format (gff) files and gene IDs downloaded from CottonFGD.

### Analysis of the conserved protein motifs and gene structure

Multiple Em for Motif Elicitation (MEME) website (http://meme-suite.org/) was used to identify the conserved protein motifs [48]. TB Tools software was used to map the evolutionary relationship, gene structure, and conserved motifs of GhNDA proteins with mast file from MEME website, nwk file from phylogenetic tree analysis, and gff3 genome file of *G. hirsutum*.

### Collinearity analysis of NDA genes in four *Gossypium* species

The complete genome sequences of four *Gossypium* species along with genome annotation files were subjected to MCScanX tool [49] to investigate the collinearity and analyze the syntenic relationship among NDA genes of four *Gossypium* species. The collinear and homologous chromosomal regions among four *Gossypium* species were visualized using Advance Circos tool in TB Tools software.

### Calculation of selection pressure

Duplicated gene pairs from four *Gossypium* species including *G. hirsutum*, *G. barbadense*, *G. arboreum*, and *G. raimondii* were identified by using MCScanX tool. The nonsynonymous (*Ka*), the synonymous (*Ks*), and *Ka*/*Ks* were calculated to investigate the selection pressure by using *Ka*/*Ks* calculator in TB Tools software.

### Gene ontology (GO) annotation analysis of *GhNDAs*

CottonFGD (https://cottonfgd.org) was used to determine the GO annotation analysis of *GhNDAs* including biological process, molecular functions and cellular component.

### Analysis of *cis*‑acting element in promoters of *GhNDAs*

The upstream sequences (2000 bp) of the NDA sequences were retrieved from the genome sequence in *G. hirsutum*. Then, the retrieved promoter sequences were submitted to PlantCARE (http://bioinformatics.psb.ugent.be/webtools/plantcare/html/) [50]. Schematic diagram of phylogenetic tree and *cis*-acting elements was drawn with the help of TB Tools.

### Expression patterns under different alkaline stress

RNA-Seq data (GSE165472) was used to analyze the expression level of differentially expressed genes (DEGs) under different alkaline stress (pH = 8.5 NaOH, 125 mM NaHCO_3_). The heat map along with phylogenetic tree and *cis*-acting elements was generated through TB Tools software by using fragments per kilo base of exon per million fragments mapped (FPKM).

### Plant materials and alkaline treatments

*Gossypium hirsutum* cv. Zhong 9807 which is obtained from the Institute of Cotton Research of CAAS was used. Cotton seeds were sown in pots containing the soil under a 16-h light/8-h dark cycle at 25 °C for approximately 30 days. Seedlings with three true leaves and one heart-shaped leaf were washed carefully and transplanted into conical flasks containing 125 mM NaHCO_3_ solution for 0 and 12 h. Leaves and root samples were collected. Each sample was replicated three times. All these samples were immediately frozen in liquid nitrogen and kept at − 80 °C for subsequent analysis.

### RNA extraction and quantitative real‑time (qRT‑PCR) analysis

The total RNA of cotton roots and leaves were extracted using EASYspin Plus Plant RNA Kit (Aidlab, Beijing, China). The quantity and quality were determined by a NanoDrop 2000 Spectrophotometer (NanoDrop Technologies, Wilmington, DE, USA). The cDNA was reverse using the EasyScript® All-in-One First-Strand cDNA Synthesis SuperMix for qPCR (One-Step gDNA Removal). The qRT-PCR was performed using the Applied Biosystems@7500 Fast instrument and TransStart Top Green qPCR SuperMix. The Actin gene was used as a control. All the operational procedures followed the manufacturer’s protocols. Statistical analysis was conducted with biological replicates with mean values of three technique replicates. 2^−△△CT^ method was used to calculate the relative FC for each sample [51]. The primers were designed using NCBI Primer-Blast tool, listed in Additional file 1: Table S1.

### Gene interaction network of the GhNDA32 protein

The GhNDA32 protein interaction network was analyzed by the STRING database (https://string-db.org/) based on the homologous gene in *Arabidopsis thaliana* [52]. Cytoscape software (version 3.7.1) was used to construct a co-expression regulation network of the GhNDA32 protein [53].

### Virus-induced gene silencing (VIGS) experiment

To verify the function of the NDA genes, we selected a highly expressed gene *GhNDA32*, pYL156: *GhNDA32* vector was constructed with the restriction enzyme cutting site BamHI and SacI. The sequences of the primer pair were detailed in Additional file 1: Table S1. The GV3101 strains carrying pYL156, pYL156: *GhNDA32*, pYL156: PDS, and pYL192 were cultured to OD600 = 1.2–1.5. Each mixture was injected into the underside of cotyledons of upland cotton material Zhong 9807. After injection, the seedlings were placed in the dark overnight, and a 16-h light / 8-h dark cycle was performed at 25 °C. When the plants injected with pYL156: PDS appeared an albino phenotype, it proved that the VIGs experiment was successful.

## Supplementary Information

**Additional file 1: Table S1.** Primer pairs used for qRT-PCR.

**Additional file 2: Table S2.** Rename of NDA genes in four *Gossypium* species and other eight species.

**Additional file 3: Table S3.** Physical properties of the GhNDAs genes.

**Additional file 4: Table S4.** Calculation of non-synonymous (Ka) to synonymous (Ks) substitution.

## Data Availability

Not applicable.

## Abbreviations

NDA: The internal NAD(P)H dehydrogenase
ND: NAD(P)H dehydrogenase
VIGS: Virus-induced gene silencing
AP: Alternative respiratory pathway
ROS: Reactive oxygen species
ETC: electron transport chain
AOX: Alternative oxidase
CottonFGD: Cotton Functional Genomic Database
Ga: *Gossypium arboreum*
Gb: *Gossypium barbadense*
Gh: *Gossypium hirsutum*
Gr: *Gossypium raimondii*
HMM: Hidden Markov Model
MWs: Molecular weights
pIs: Isoelectric points
MEME: Multiple Em for Motif Elicitation
*Ka*: The nonsynonymous
*Ks*: The synonymous
GO: Gene ontology
DEGs: Differentially expressed genes
FPKM: Fragments per kilo base of exon per million fragments mapped
qRT‑PCR: quantitative real‑time PCR
NJ: Neighbor-joining
ABA: Abscisic acid
MeJA: Methyl jasmonate
GA: Gibberellin
CK: No infection
SAS: 125 mM NaHCO_3_
AS: pH = 8.5 NaOH
