# Supplementary material for: Molecular structures and functional exploration of NDA family genes respond tolerant to alkaline stress in Gossypium hirsutum L
Source: Biol Res. 2022 Jan 21;55:4. doi: 10.1186/s40659-022-00372-8 (PMC8781182; doi:10.1186/s40659-022-00372-8)
Supplement: Supplementary file 1 — Additional file 1: Table S1. Primer pairs used for qRT-PCR. [file 40659_2022_372_MOESM1_ESM.docx]

**Table S1** Primer pairs used for qRT-PCR

| Gene ID | Primer pairs for qRT-PCR (5'-3') | |
| --- | --- | --- |
| *Actin* | ATCCTCCGTCTTGACCTTG | TGTCCGTCAGGCAACTCAT |
| *GhNDA1* | GAAGGGTGGAACACGTTGAC | GATTTCGGGTTGCTGTCTCC |
| *GhNDA7* | AACAGCTGCACAACTTCCAG | GACTTGCTTGCTTGCGTAGA |
| *GhNDA8* | AATGGTTGCGTGTACCTTCG | TCCTCTTTCGCCATCCTGTT |
| *GhNDA10* | AACCAAGGGTGGTGGTGTTA | GCGAGACACAAACGATGTCA |
| *GhNDA11* | TCACCCTTATCGACCCGAAG | CCGTGATATTTGTGGCAGCA |
| *GhNDA14* | TGGTGGAAGGCCTCTTACAG | AAGTGGCTACATCACCCACA |
| *GhNDA19* | GAGGAGTCACCTCAGGCAAT | CCTTCGACCAGTTGCAAACA |
| *GhNDA22* | AGACAAGAGCTCCTGGCAAT | AGCTTCTTGGTGCCCATAGT |
| *GhNDA25* | CTTCGGATGGGAGCCAAGTA | TTATTGCGACCCTTCACCCT |
| *GhNDA30* | CCTTGGGCAATTTGCTCCTT | GTGCTACGACCCATGGAAAC |
| *GhNDA31* | AACAGCTGCACAACTTCCAG | GACTTGCTTGCTTGCGTAGA |
| *GhNDA32* | GGAGTGGAGTTCAGTGGTGA | TGAACACCCGACTTCCTCAA |
| *GhNDA34* | TCTCTAGCAGGCTTCGTCAG | TGAGCCAGTTCATAGCCACA |
| *GhNDA35* | CATGCCATGGTAGTTGCGAA | GGGCGATTGCATCTTTCCTT |
| *GhNDA40* | GCTGTAAAGGCCATCAAGGG | CGGTGATTGTGGGTTGTTGT |
| *GhNDA48* | AGACAAGAGCTCCTGGCAAT | AGCTTCTTGGTGCCCATAGT |
| *GhNDA50* | CAAAGGCTGCTGCTAGACTG | TTCTCCAGCAGCACTTACGA |
| *GhNDA51* | AAGGTGGAACCGTCATTTGC | CACTATGCCCAGTGGCAATC |
| pYL156: *GhNDA32* | AAGGCCTCCATGGGGATCCC  CTCAATGGGCTT | TCGAGACGCGTGAGCTCGCA  ATTACAAGCTT |
